# Research on the evaluation of emergency management capability for urban public health emergencies under the perspective of resilience—a case study of Henan Province, China

**DOI:** 10.3389/fpubh.2024.1431158

**Published:** 2024-11-04

**Authors:** Yu Hao, Yuxin Tie, Lijun Zhang, Fan Zhang, Chaolun Sun

**Affiliations:** ^1^Safety and Emergency Management Research Center, Henan Polytechnic University, Jiaozuo, China; ^2^Laboratory of Emergency Management, Henan Polytechnic University, Jiaozuo, China; ^3^School of Information Management, Central China Normal University, Wuhan, China

**Keywords:** public health emergencies, the full process balance of emergency management, emergency management capacity, capacity evaluation, TOPSIS model

## Abstract

With the rapid development of the economy and society, the likelihood of sudden public health emergencies in urban areas continues to rise. In particular, major infectious diseases that have gained global attention, such as the SARS virus, H1N1 influenza, Ebola outbreak, and COVID-19 pandemic, have presented significant challenges to urban emergency management systems. Evaluating emergency management capability is a fundamental requirement for developing emergency response capacity. To this end, this study combines the theory of resilience with the theory of full-process equilibrium emergency management, selects 31 evaluation indicators from six key aspects: preparedness, forewarning, mitigation, disposal, recovery, and learning. The indicator weights are determined using the AHP-Entropy Weight Method, and a TOPSIS model is constructed to assess the emergency management capability of urban public health emergencies. The model’s applicability is validated by examining 18 cities in Henan Province. The findings suggest that Jiaozuo, Hebi, Zhengzhou, and Luohe possess relatively robust emergency management capabilities for sudden public health incidents, whereas Kaifeng, Pingdingshan, and Shangqiu exhibit weaker capabilities.

## 1 Introduction

The public security situation has become increasingly severe with the rapid development of China’s economy and society. A series of sudden public health emergencies, including SARS, H1N1 influenza, and the COVID-19 pandemic, have emerged one after another (1). These crises not only pose a severe threat to the life and health of the population but also impact economic development, social stability, and political security. Additionally, they present a significant challenge to the emergency management capabilities of urban areas. The emergence of COVID-19, a novel coronavirus causing pneumonia, was sudden at the end of 2019. Its rapid spread prompted the World Health Organization (WHO) to declare it a Public Health Emergency of International Concern (PHEIC) in January 2020 (2). COVID-19 is the most severe global pandemic of infectious diseases in a century (3). It presents the most challenging public health emergency in China since the founding of the People’s Republic due to its rapid and difficult-to-control spread (4).

At present, China is in the period of social transformation, which is an important period to improve the public health emergency response system and consolidate the emergency response capacity in an all-round way. Emergencies refer to natural disasters, accident disasters, public health events and social security events that occur suddenly, cause or may cause serious social harm and need to be dealt with by emergency measures (5). The public health emergencies studied in this paper are characterized by the inability to measure with tools, the difficulty to judge the signs of its occurrence before it occurs, and the rapid outbreak, wide radiation and will have a bad impact on the urban economic and social order, which also poses a challenge to the construction of resilient cities. Therefore, improving the emergency management ability of urban public emergencies is the only way to improve the urban comprehensive emergency system and capacity modernization (6), and it is also an important pillar of the construction and development of resilient cities (7).

**Figure 1:**
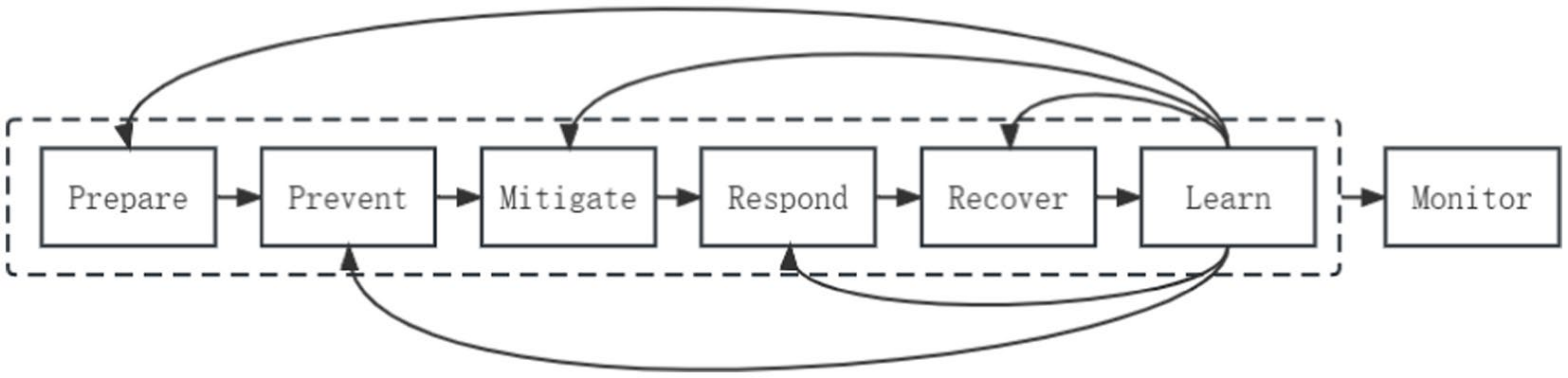
The full process of emergency management is based on a balanced “6 + 1” mechanism.

Throughout the emergency management process, any critical step overlooked or any present loophole can result in serious errors, leading to irreparable loss of life, property, and social disorder. This sensitivity requires balance throughout the entire process. In 2020, Professor Zhang (8) proposed a new issue of balanced emergency management for the full process mechanism in China, addressing the imbalance problem. He suggests that “the full process balance of emergency management” in China includes six hierarchical mechanisms and one cross-stage mechanism, refining emergency management, risk management, and crisis management into preparation and response, prevention and mitigation, recovery and learning, with monitoring running through the entire emergency management process, as illustrated in Figure 1.

Therefore, from the perspective of the whole process equilibrium of emergency management, this paper constructs the evaluation index system of emergency management ability of urban public health emergencies, and uses TOPSIS method to evaluate the level of urban emergency management, which provides qualitative evaluation basis for the government to make emergency management decisions. In addition, it also puts forward operational countermeasures and suggestions for the emergency management ability of cities in response to public health emergencies, aiming at providing policy suggestions for the development of urban emergency management.

## 2 Literature review

In 1997, the Federal Emergency Management Agency (FEMA) and the National Emergency Management Association (NEMA) conducted research. They released the Capability Assessment for Readiness (CAR) framework to evaluate the disaster preparedness of states and localities. This framework comprised 13 primary indicators, 203 secondary indicators, and 1,014 tertiary indicators (9). In the year 2000, the United States refined, improved, and revised this evaluation system (10). Since the terrorist attacks 9/11, the field of emergency management has rapidly developed and established its system in both practice and research, leading to a significant increase in the evaluation of emergency management capabilities. Japan has collaborated with government agencies to establish a collaborative disaster prevention capability evaluation system, which includes indicators for crisis assessment, disaster management, material support, and information communication to evaluate regional disaster prevention capabilities (11).

Regarding theoretical research, Miguel (12) employed the Fuzzy Analytic Hierarchy Process (FAHP) to identify performance evaluation indicators for emergency decision-making in emergency management departments. He developed a multi-criteria decision model (MCDM) to enhance public emergency decision-making and response capabilities. Guofeng (13) proposed a Capability Maturity Model (FE-CMM) for building fire emergency management, which is based on building fire threats and integrated with the fire control center to achieve pre-control and exemplary management of building fires. Arleiny (14) conducted qualitative research to analyze the causes of fire disasters in Indonesia and evaluated the search and rescue capabilities of different regions in the event of fire disasters through case studies.

Although China’s research on evaluating emergency management capabilities for sudden events started relatively late, it has accumulated a wealth of research results. Cheng et al. (15) developed a comprehensive city emergency management capability evaluation system from a safety development perspective, utilizing a combination of the matter-element extension model and cloud model. Chuai et al. (16) determined indicator weights through the AHP-entropy method and constructed a disaster emergency capability evaluation model to assess seven geographical regions, 31 provinces, and 339 cities in China. Jia et al. (17), Ming et al. (18), Liu et al. (19), and Huang et al. (20) conducted detailed evaluations of earthquake emergency capabilities in the island, Hainan, Xinjiang, and Guangdong regions, respectively.

International research on evaluating emergency management capacity for sudden events is still in its infancy and needs a robust theoretical framework. The United States has taken the lead in early evaluations of emergency management capacity for sudden events worldwide, and its comprehensive evaluation index system serves as a reference for many countries researching emergency management capacity evaluation. While there have been numerous research achievements on evaluating emergency management capacity for sudden events both domestically and internationally, they have primarily focused on natural disasters or safety accidents, with a heavy emphasis on single natural disaster events or single disposal stages such as hazardous chemicals, earthquakes, and floods. There need to be more relevant evaluation studies on sudden public health events, with most studies focusing on the past two years and still in the preliminary stages. The research on evaluating emergency management capacity for the entire process of sudden events is relatively scarce and urgently requires improvement. Based on this, this paper uses subjective analytic hierarchy process, objective entropy weight method and TOPSIS method to process the data, evaluates the emergency management ability of urban public health emergencies in 18 cities of Henan Province, and puts forward countermeasures and suggestions to improve the emergency management ability, in order to provide qualitative evaluation tools for the government to carry out emergency management efficiently and immediately.

## 3 Building a public health emergency in the city emergency management capacity evaluation model

**Figure 2:**
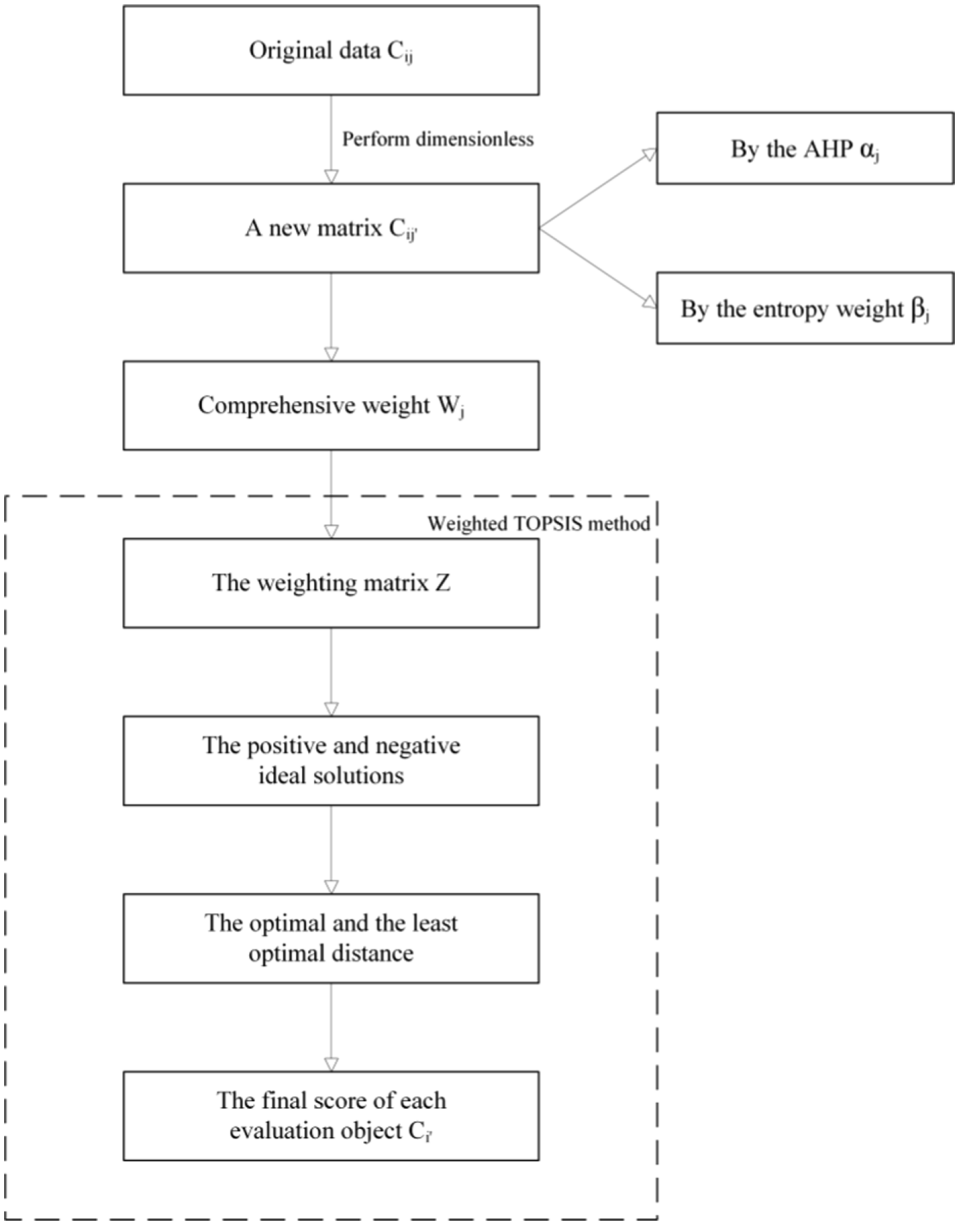
The computational flowchart for the evaluation model.

By constructing an evaluation model for the emergency management capability of urban public health emergencies, this study utilizes the TOPSIS method to compute the scores of the evaluation objects. In calculating the comprehensive weights of the evaluation indicators, a combination of the Analytic Hierarchy Process (AHP) and entropy weight method is employed. AHP and the entropy weight method represent subjective and objective weighting approaches, respectively. The weights obtained through subjective weighting methods are influenced by human subjectivity, while objective methods may fail to accurately reflect the evaluator’s emphasis on different indicators, potentially leading to discrepancies with the actual conditions. Consequently, this study adopts a combined approach of AHP and entropy weighting, effectively addressing the subjective biases introduced by human factors and the objective shortcomings of overlooking indicator characteristics, thereby resulting in a more scientifically rational assignment of indicator weights. The computational flowchart for the evaluation model is illustrated in Figure 2.

### 3.1 The setting of the index system

Urban resilience is the ability of cities to quickly return to normal operation after responding to emergencies. It is the proper meaning of building a holistic urban safety system, and urban public health event response capacity building is an important part of it (21). This paper adheres to the focus of resilient urban development. In order to comprehensively enhance the city’s ability to cope with the complex social risks brought by public emergencies (22), based on the “6 + 1” model of the whole process equilibrium theory of emergency management, the emergency management ability of urban public health emergencies is divided into six aspects: preparation ability, early warning ability, mitigation ability, disposal ability, recovery ability and learning ability. Among them, the ‘monitoring’ stage in the whole process equilibrium theory of emergency management should not be regarded as a stage alone because it involves many key links, even the whole process of emergency management. Therefore, we can build a truly safe and reliable ‘resilient city ‘with more refinement and specialization.

The selection of evaluation indicators follows the principles of indicator independence, scientificity, and data availability and draws on relevant research results (23–30). In light of the current COVID-19 epidemic situation in China, and fully considering the capacity requirements of resilient city construction, using the Delphi expert method to screen the indicators, members of the Ministry of Emergency Management’s think tank were selected as experts to score the selected evaluation indicators, and, based on the scoring, the mean value of the scores of the indicators was calculated, and the indicators with an importance level of 4.0 or less were removed. A corresponding evaluation indicator system is constructed based on this framework, as shown in Table 1.

**Table 1:** Evaluation index system and weight of urban public health emergency management ability.

Goal	Domain/weight	Indicator names	No.	AHP weight	EMW weight	Combined weight	Literature sources
Urban Public Health Emergency Management Capacity (A)	Preparedness capability (B1) 0.2152	Proportion of government financial input on public security	C1	0.0393	0.0336	0.0385	(23, 24, 27, 30)
		Proportion of government financial input to education	C2	0.0235	0.0265	0.0264	
		Proportion of government financial input on medical and health care	C3	0.0270	0.0228	0.0264	
		Proportion of government expenditure on transport	C4	0.0159	0.0302	0.0232	
		Proportion of government financial input in grain and oil reserves	C5	0.0301	0.0443	0.0387	
		Proportion of government financial input on disaster prevention and emergency management	C6	0.0274	0.1240	0.0619	
	Forewarning capability (B2) 0.1295	Mobile phone penetration	C7	0.0253	0.0094	0.0164	(23, 24, 27, 30)
		Fixed-line telephone penetration	C8	0.0145	0.0169	0.0166	
		Television coverage	C9	0.0175	0.0191	0.0194	
		Broadcast coverage	C10	0.0107	0.0109	0.0115	
		Comprehensive water supply capacity per 10,000 population per day	C11	0.0196	0.0351	0.0278	
		Urban water access rate	C12	0.0198	0.0113	0.0159	
		Urban gas penetration rate	C13	0.0362	0.0119	0.0220	
	Mitigation capability (B3) 0.1306	Density of social and civil welfare institutions providing accommodation	C14	0.0583	0.0354	0.0482	(24, 25, 28, 30)
		Number of beds in social and civil affairs welfare institutions	C15	0.0400	0.0436	0.0443	
		*Per capita* civilian automobile ownership	C16	0.0297	0.0433	0.0380	
	Disposal capability (B4) 0.2398	Health facilities per 10,000 population	C17	0.0644	0.0172	0.0353	
		Health technicians per 10,000 population	C18	0.0621	0.0346	0.0492	
		Beds in medical and health institutions per 10,000 population	C19	0.0686	0.0425	0.0573	
		Self-sufficiency in food per 10,000 population	C20	0.0376	0.0193	0.0286	
		Self-sufficiency of oil per 10,000 population	C21	0.0301	0.0631	0.0462	
		Self-sufficiency of meat per 10,000 population	C22	0.0238	0.0202	0.0232	
	Recovery capability (B5) 0.2003	Proportion of government expenditure on social security and employment	C23	0.0396	0.0152	0.0261	(23, 26)
		Public revenue per 10,000 population	C24	0.0269	0.0388	0.0343	
		*Per capita* disposable income	C25	0.0200	0.0256	0.0240	
		*Per capita* GDP	C26	0.0207	0.0468	0.0330	
		Proportion of working population	C27	0.0200	0.0397	0.0299	
		Proportion of the number of people insured by unemployment insurance	C28	0.0244	0.0434	0.0345	
		Proportion of the number of people insured by basic medical insurance	C29	0.0271	0.0113	0.0186	
	Learning capability (B60.0846	Number of public health emergencies	C30	0.0302	0.0147	0.0223	(24–26)
		Number of disaster prevention and mitigation trainings	C31	0.0699	0.0492	0.0623	

### 3.2 Calculation of evaluation model

#### 3.2.1 The original matrix of dimensionless data is established

Based on the original data, the spatial matrix C comprises n cities and m indexes.


$$C={\left[C_{ij}\right]}_{n*m};i=1,2,3,\ldots ,n;j=1,2,3,\ldots ,m$$

Next, the standardization of data processing.

Positive indicators:


$$C_{ij}^{\prime }=\frac{C_{ij}-MIN\left(C_{j}\right)}{\mathrm{MAX}\left(C_{j}\right)-MIN\left(C_{j}\right)}+0.0001$$

Negative indicator:


$$C_{ij}^{\prime }=\frac{\mathrm{MAX}\left(C_{j}\right)-C_{ij}}{\mathrm{MAX}\left(C_{j}\right)-MIN\left(C_{j}\right)}+0.0001$$

Getting a new matrix:


$$C^{\prime }={\left[C_{ij}^{\prime }\right]}_{n*m};i=1,2,3,\ldots ,n;j=1,2,3,\ldots ,m$$

#### 3.2.2 Determine the comprehensive weight of evaluation indicators

The Analytic Hierarchy Process (AHP) and Entropy Weight Method (EWM) represent subjective and objective weighting methods. AHP determines indicator weights by comparing their relative importance on a 1–9 scale (31), while EWM quantifies indicator weights by calculating entropy values based on collected data (32, 33). The indicator weights obtained by subjective weighting methods are subject to human subjectivity. In contrast, objective weighting methods cannot reflect the evaluators’ degree of importance for different indicators, and may even result in opposite situations to the actual indicators. Therefore, this study adopts a combination of AHP-EWM to effectively address the subjective influence caused by human factors and the objective deficiency of ignoring indicator characteristics, thus making the indicator weight values more scientifically reasonable. Comprehensive weight calculation formula:


$$W_{j}=\frac{\sqrt{\alpha_{j}\times \beta_{j}}}{\sum_{j=1}^{n}\sqrt{\alpha_{j}\times \beta_{j}}}$$

Where: $\alpha_{j}$ is the weight calculated by the analytic hierarchy process; $\beta_{j}$ is the weight calculated by the entropy weight method.

#### 3.2.3 Weighted TOPSIS method

The Technique for Order Preference by Similarity to Ideal Solution (TOPSIS) method is widely used for multi-objective analysis of final solutions in systems engineering, with applications in various fields. The method involves constructing a space based on a normalized initial data matrix, where the final solution’s positive and negative ideal solutions define the boundaries. The evaluated solution is represented as a point in this space, and its relative closeness to the ideal solutions is determined by calculating the distance between the point and the positive and negative ideal solutions. The quality of the solution is evaluated based on the size of the distance.

First, calculate the weighting matrix.


$$Z=A^{\prime }*W_{j}={\left[z_{ij}\right]}_{i*j}$$

In the formula, $A^{\prime }$ is the standardized judgment matrix after translation, and $W_{j}$ is the final combined weight of each index.

Secondly, the positive and negative ideal solutions are calculated.

Positive ideal solution:


$$Z^{+}=\left(Z_{1}^{+},Z_{2}^{+},\ldots ,Z_{i}^{+}\right)=\left(\max\left\{z_{i1}\right\},\max\left\{z_{i2}\right\},\ldots ,\max\left\{z_{ij}\right\}\right)$$

Negative ideal solution:


$$Z^{-}=\left(Z_{1}^{-},Z_{2}^{-},\ldots ,Z_{i}^{-}\right)=\left(\min\left\{z_{i1}\right\},\min\left\{z_{i2}\right\},\ldots ,\min\left\{z_{ij}\right\}\right)$$

To get the optimal distance:


$$D_{i}^{+}=\sqrt{\overset{m}{\underset{j=1}{\sum }}{\left(Z_{j}^{+}-z_{ij}\right)}^{2}}$$

The least optimal distance:


$$D_{i}^{-}=\sqrt{\overset{m}{\underset{j=1}{\sum }}{\left(Z_{j}^{-}-z_{ij}\right)}^{2}}$$

After that, the relative closeness between each evaluation object and the ideal solution is calculated:


$$C_{i}=\frac{D_{i}^{-}}{D_{i}^{+}+D_{i}^{-}}$$

Finally, the evaluation results are standardized to obtain the final score of each evaluation object:


$$C_{i}^{\prime }=\frac{C_{i}-MIN\left(C_{i}\right)}{\mathrm{MAX}\left(C_{i}\right)-MIN\left(C_{i}\right)}$$

The evaluation result of the evaluation object can be divided into five levels according to the equal interval: high, medium-high, medium, medium-low, and low.

## 4 An empirical study and analysis of emergency management capability evaluation—a case study of Henan Province

### 4.1 Data source and data processing

The data for this article was originally sourced from the “2021 Statistical Yearbook” and “2021 National Economic and Social Development Statistical Bulletin” of 17 prefecture-level cities and one provincial-level administrative division (also known as a provincial-level county-level city) in Henan Province, as well as the official websites of the Henan Provincial Bureau of Statistics and various municipal statistical bureaus. The specific evaluation objects are listed in Table 2. The collected raw data underwent dimensionless processing, and the AHP-Entropy Weight Method was utilized to assign weights to the evaluation indicators, with weight results in Table 1. Finally, the TOPSIS method was employed to compare the relative closeness of each evaluation object to the ideal solution and obtain the evaluation value. The relative closeness and comprehensive evaluation ranking of the emergency management capacity of 18 evaluation objects in Henan Province for public health emergencies are displayed in Table 3. The evaluation values and rankings of the six primary indicators are presented in Table 4.

**Table 2:** Urban public health emergency management capability evaluation object.

Province	Evaluation object					
Henan Province	Zhengzhou	Kaifeng	Luoyang	Pingdingshan	Anyang	Hebi
	Xinxiang	Jiaozuo	Puyang	Xuchang	Luohe	Sanmenxia
	Nanyang	Shangqiu	Xinyang	Zhoukou	Zhumadian	Jiyuan

**Table 3:** Evaluation of comprehensive performance of public health emergency management capacity in Henan Province.

City	D+	D-	$C_{i}$	$C_{i}^{\prime }$	Ranking
Zhengzhou	0.1321	0.1224	0.0674	0.9644	3
Kaifeng	0.1430	0.0698	0.0459	0.1337	16
Luoyang	0.1260	0.1004	0.0622	0.7620	5
Pingdingshan	0.1454	0.0673	0.0443	0.0711	17
Anyang	0.1420	0.0809	0.0508	0.3234	14
Hebi	0.1226	0.1143	0.0676	0.9743	2
Xinxiang	0.1350	0.0753	0.0502	0.2982	15
Jiaozuo	0.1206	0.1146	0.0683	1.0000	1
Puyang	0.1316	0.0977	0.0597	0.6671	6
Xuchang	0.1382	0.0838	0.0529	0.4034	11
Luohe	0.1221	0.1034	0.0643	0.8436	4
Sanmenxia	0.1362	0.0905	0.0559	0.5210	8
Nanyang	0.1430	0.0920	0.0549	0.4791	9
Shangqiu	0.1586	0.0690	0.0425	0.0000	18
Xinyang	0.1377	0.0961	0.0576	0.5856	7
Zhoukou	0.1479	0.0850	0.0511	0.3343	12
Zhumadian	0.1508	0.0858	0.0508	0.3234	13
Jiyuan	0.1457	0.0899	0.0535	0.4258	10

**Table 4:** Evaluation of 6 first-level indexes in 18 cities of Henan Province.

City	B1 preparedness capability	Ranking	B2 forewarning capability	Ranking	B3 mitigation capability	Ranking
Zhengzhou	0.1697	14	0.0121	17	0.6335	5
Kaifeng	0.1092	17	0.4397	8	0.3732	10
Luoyang	0.1180	16	0	18	1	1
Pingdingshan	0.3815	11	0.1559	13	0.3722	11
Anyang	0.6523	3	1	1	0.1828	15
Hebi	1	1	0.6704	6	0.6609	3
Xinxiang	0.5533	6	0.4103	9	0.4676	9
Jiaozuo	0.6204	5	0.8533	3	0.9678	2
Puyang	0.8614	2	0.9448	2	0.3027	13
Xuchang	0.4233	10	0.4727	7	0.6036	6
Luohe	0	18	0.6907	5	0.5776	7
Sanmenxia	0.2970	12	0.3753	10	0.1653	16
Nanyang	0.6279	4	0.0754	16	0.0466	17
Shangqiu	0.5477	7	0.0796	14	0.2313	14
Xinyang	0.2665	13	0.3210	11	0.4760	8
Zhoukou	0.5448	8	0.0762	15	0.6421	4
Zhumadian	0.4382	9	0.2683	12	0	18
Jiyuan	0.1314	15	0.8169	4	0.3171	12

City	B4 disposal capability	Ranking	B5 recovery capability	Ranking	B6 learning capability	Ranking
Zhengzhou	0.8326	3	1	1	0.2177	10
Kaifeng	0.4041	9	0.0184	16	0.1851	13
Luoyang	0.5314	7	0.4829	4	0.2852	9
Pingdingshan	0.0350	16	0.2923	9	0.1232	15
Anyang	0.3762	10	0.0484	14	0	18
Hebi	0.2686	11	0.3236	7	0.6251	3
Xinxiang	0.2589	12	0.1417	11	0.2889	8
Jiaozuo	0.6704	6	0.5825	3	0.1157	16
Puyang	0.5069	8	0.0008	17	0.3311	7
Xuchang	0	18	0.3347	6	0.4366	5
Luohe	0.7634	4	0.3188	8	0.8455	2
Sanmenxia	0.8527	2	0.4747	5	0.3689	6
Nanyang	1	1	0.1429	10	0.1502	14
Shangqiu	0.0308	17	0.0408	15	0.0431	17
Xinyang	0.1631	14	0.0736	13	1	1
Zhoukou	0.0789	15	0	18	0.5617	4
Zhumadian	0.7347	5	0.0930	12	0.2012	11
Jiyuan	0.2483	13	0.7984	2	0.1863	12

### 4.2 Analysis of emergency management ability of urban public health emergencies in Henan Province

**Figure 3:**
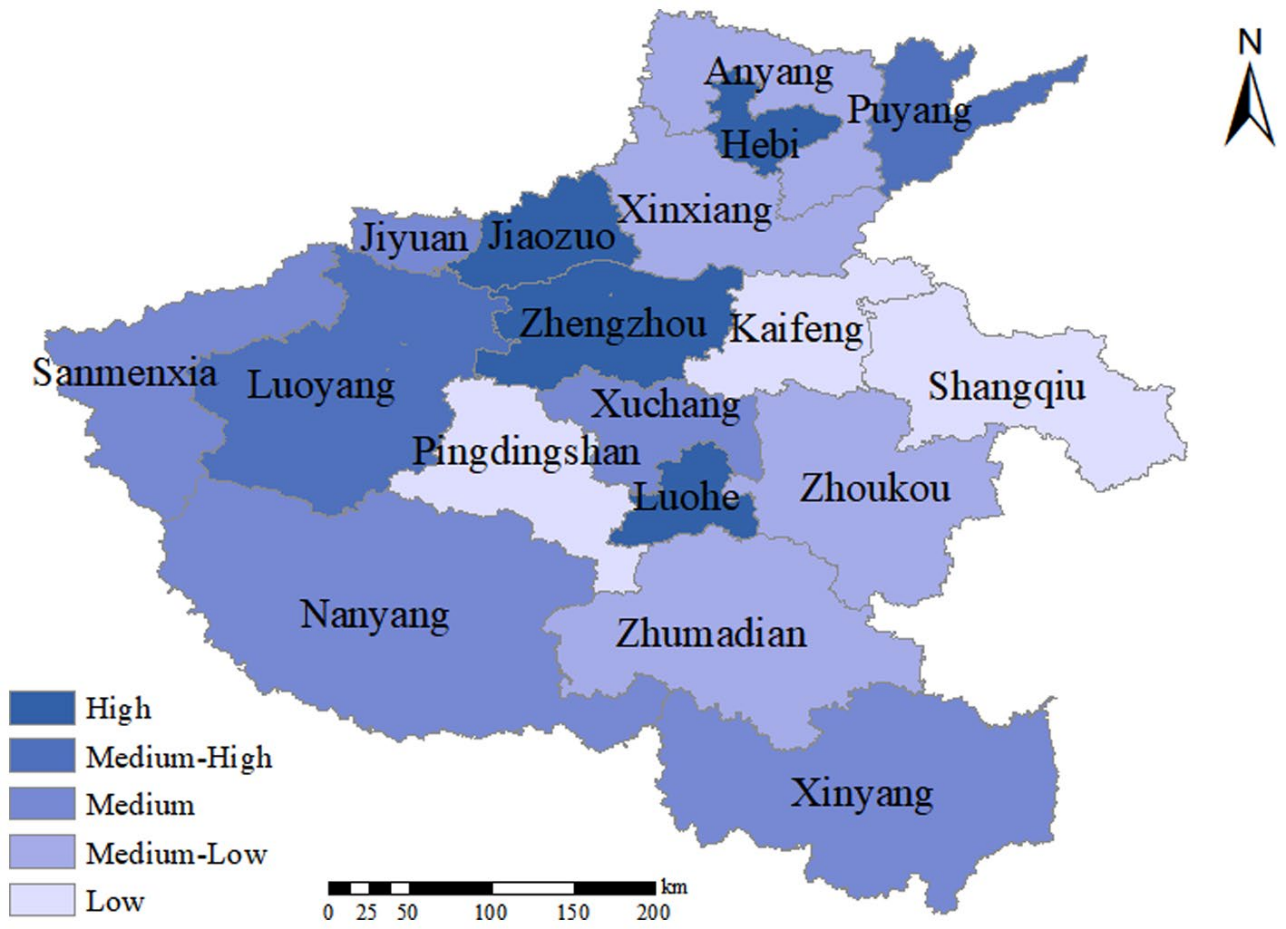
Public health emergencies emergency management capacity distribution of Henan Province.

As depicted in Figure 3, the emergency management capabilities of cities in Henan Province in response to sudden public health incidents can be classified into five levels based on their rankings, as presented in Table 5. The first tier, led by Jiaozuo City, exhibits a slightly higher level of medical infrastructure development compared to other cities of the same level within Henan Province. Additionally, a higher proportion of public safety expenditure in general public budget expenditures is also one of its significant influencing factors. Hebi City, Zhengzhou City, and Luohe City also belong to the first tier. Zhengzhou City, the provincial capital of Henan Province, has a large permanent population and is situated in a transportation hub position, with many population movements, making personnel management more challenging. However, the quality of medical care in Zhengzhou has improved in recent years due to the development of high-tech industries, digital management models, and urban logistics and social security. Moreover, Zhengzhou has a more vital ability to attract and accommodate talents. Nevertheless, the COVID-19 pandemic and the “7.20” major flood have significantly impacted Zhengzhou’s economic and management stability as the provincial capital city. Therefore, in the comprehensive evaluation, Zhengzhou’s relative closeness is greatly influenced by its pre-event preparation and event warning capabilities. Its emergency management capabilities for sudden public health incidents rank third in the province, following Jiaozuo city and Hebi city.

**Table 5:** Echelon division of public health emergency management capacity in Henan Province.

Category	City
First echelon	Jiaozuo, Hebi, Zhengzhou, and Luohe
Second echelon	Luoyang and Puyang
Third echelon	Xinyang, Sanmenxia, Nanyang, Jiyuan, and Xuchang
Fourth echelon	Zhoukou, Zhumadian, Anyang, and Xinxiang
Fifth echelon	Kaifeng, Pingdingshan, and Shagnqiu

Hebi and Luohe cities have demonstrated exceptional preparedness, mitigation, and learning capabilities throughout the emergency management process for sudden public health incidents in Henan Province. This has been achieved through the establishment of comprehensive emergency plans, the construction of emergency command centers and material reserve centers, and the implementation of emergency drills and training measures. These efforts have provided robust support for responding to sudden public health incidents. By strengthening epidemic monitoring and early warning systems, timely isolation, investigation, and tracking measures have been implemented, effectively curbing the spread of the epidemic and reducing its risk. Through the timely identification of problems and the analysis of epidemic prevention and control work, valuable experiences have been accumulated, and emergency plans and response mechanisms have been continuously improved and enhanced. These efforts have laid a solid foundation for future responses to sudden public health incidents.

Based on the evaluation data, it is evident that the emergency management capacity for sudden public health incidents in most cities in Henan Province is concentrated in the third and fourth tiers. This phenomenon suggests substantial disparities in emergency management capacity for sudden public health incidents among cities in Henan Province, with over half of the cities requiring improvement in emergency management capacity. A holistic perspective reveals the urgent need to enhance emergency management capacity in these cities.

### 4.3 Evaluation and analysis of urban public health emergency management ability in 6 stages in Henan Province

#### 4.3.1 Preparedness capability

**Figure 4:**
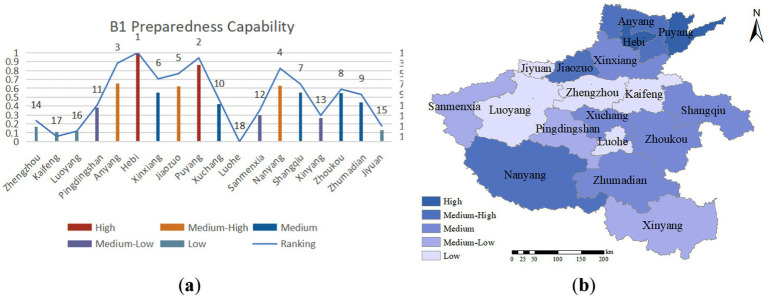
**(A)** Preparedness capability evaluation value and ranking; **(B)** preparedness capability assessment distribution.

Emergency preparedness refers to a city’s ability to proactively plan and allocate resources to minimize the impact of a public health emergency. Effective emergency preparedness involves careful consideration of potential scenarios and integrating and coordinating necessary resources. As illustrated in Figure 4, northern Henan Province exhibits a relatively high level of emergency preparedness, with Hebi, Puyang, Anyang, Nanyang, and Jiaozuo ranking at the forefront of public health emergency preparedness evaluations. This can be attributed to the significant government expenditure allocated towards public safety, medical and healthcare, disaster prevention, and emergency management within the general public budget.

#### 4.3.2 Forewarning capability

**Figure 5:**
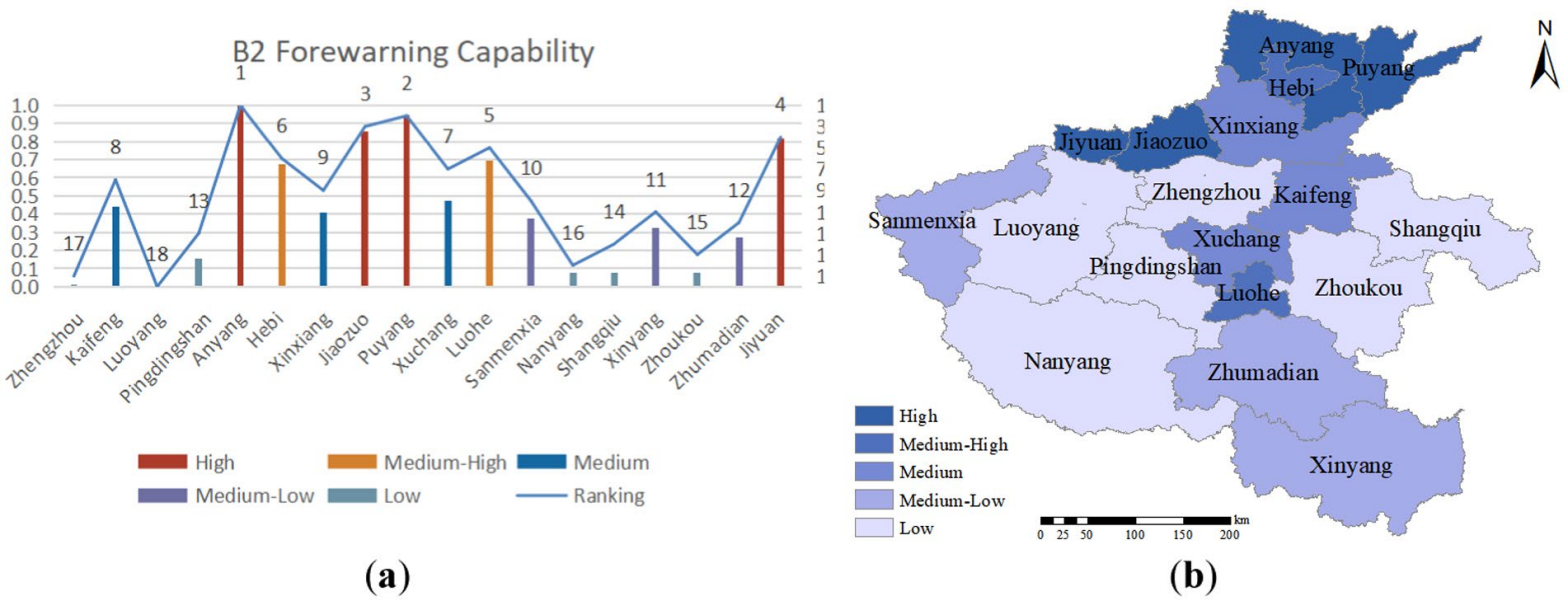
**(A)** Forewarning capability evaluation value and ranking; **(B)** forewarning capability assessment distribution.

The ability to issue emergency warnings involves predicting and alerting potential crises through various means and technologies before their occurrence, and promptly notifying relevant departments and personnel for emergency response, thereby minimizing harm and losses caused by emergencies. Warning capability is a critical component of emergency management for public health crises, and its accuracy and timeliness play a vital role in crisis resolution. As illustrated in Figure 5, the warning capability in the northern Henan region surpasses that of other areas, with Anyang, Puyang, and Jiaozuo ranking high in the evaluation. This is attributed to the region’s high communication penetration rate and extensive coverage of television and radio broadcasting.

#### 4.3.3 Emergency capability

**Figure 6:**
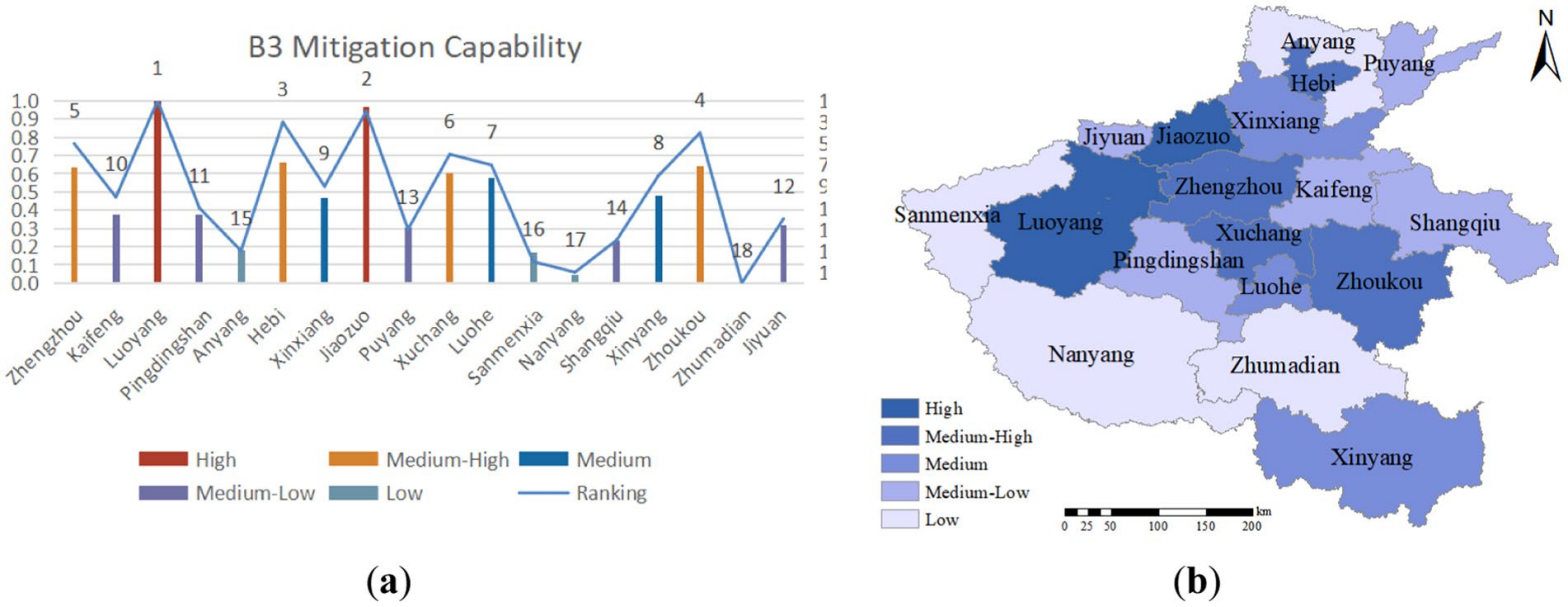
**(A)** Emergency capability evaluation value and ranking; **(B)** emergency capability assessment distribution.

Emergency mitigation capacity is the ability to reduce or eliminate potential risks and hazards in an emergency. Urban healthcare systems can better respond to public health emergencies and minimize losses by mitigating risks. As depicted in Figure 6, the central region of Henan Province exhibits a relatively strong emergency mitigation capacity, while Zhoukou and Nanyang exhibit relatively weak capacities. Despite having many social welfare institutions providing accommodation, the large urban areas and high population densities of these two cities make it challenging for these institutions to effectively share the burden on the healthcare system during a public health emergency, resulting in a lower emergency mitigation capacity.

#### 4.3.4 Disposal capability

**Figure 7:**
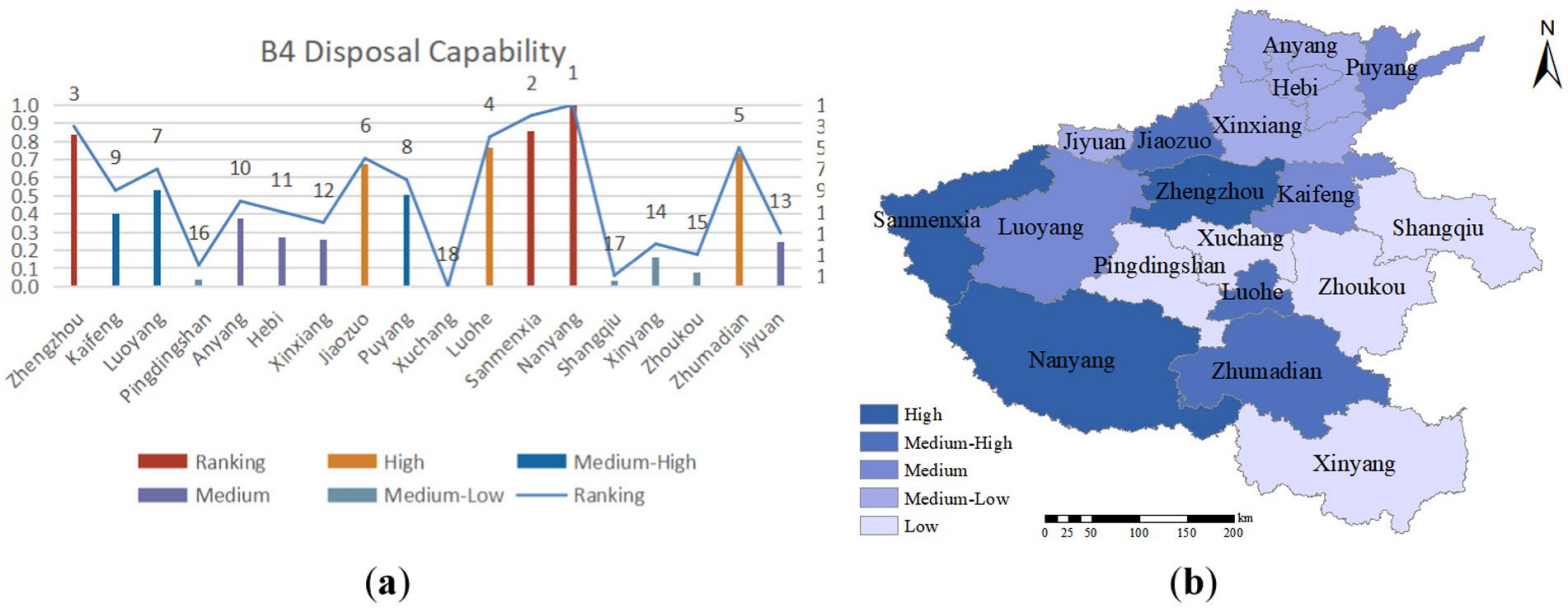
**(A)** Disposal capability evaluation value and ranking; **(B)** disposal capability assessment distribution.

Emergency response capability is the capacity to promptly and effectively address and rescue individuals during emergencies or disasters. In an unexpected public health crisis, the medical and health standards per 10,000 individuals and the self-sufficiency of essential food items such as grain, oil, and meat can bolster a city’s ability to withstand such crises. As illustrated in Figure 7, urban areas in Henan Province exhibit a slightly higher overall emergency response capability, with Nanyang City serving as a significant population and grain hub in the province. Situated in a central region with convenient transportation, Nanyang City enjoys a strategic location advantage, connecting North China, East China, South China, and other regions, facilitating swift response and management of sudden public health emergencies.

#### 4.3.5 Recovery capability

**Figure 8:**
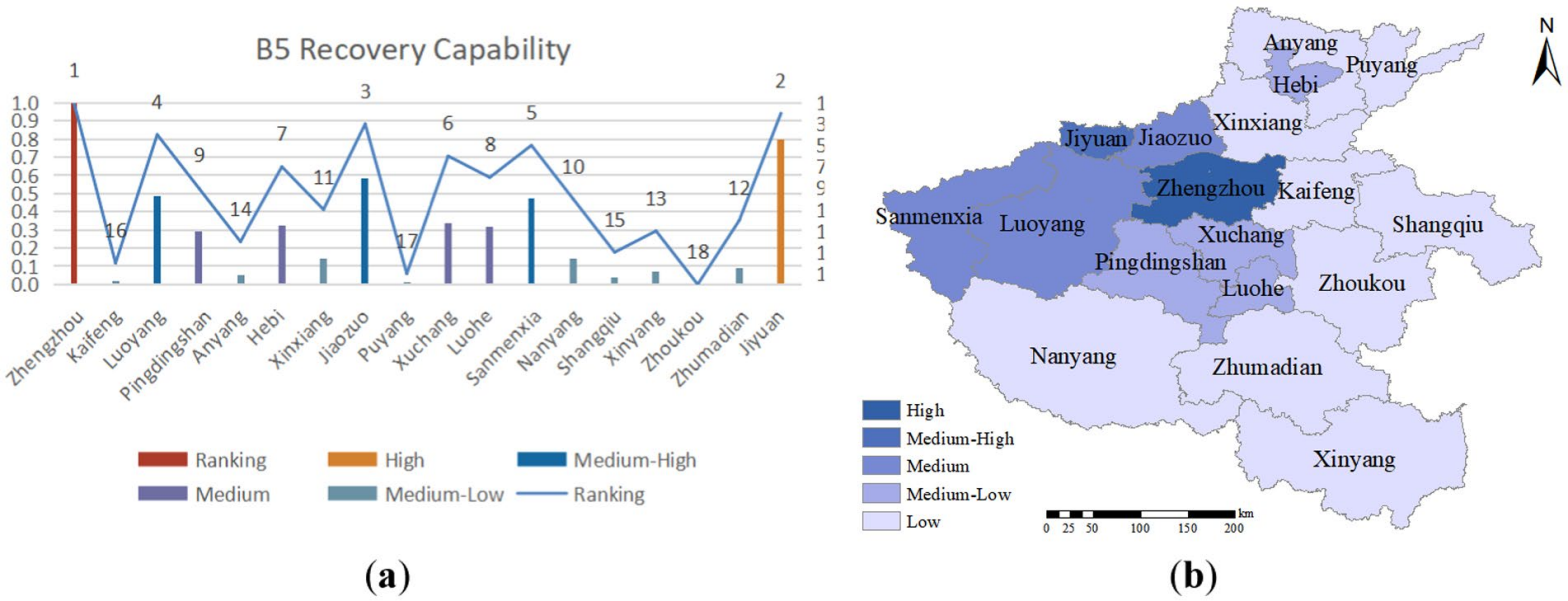
**(A)** Recovery capability evaluation value and ranking; **(B)** recovery capability assessment distribution.

Emergency recovery capacity refers to the ability to restore normal social and economic activities swiftly and public order following a public health emergency through measures such as supporting economic reconstruction, ensuring social stability, and facilitating the resettlement of affected residents and businesses. As illustrated in Figure 8, the western and central regions of Henan Province exhibit more robust post-emergency recovery capabilities, followed by the southern part. At the same time, the eastern and northern areas display relatively weaker post-emergency recovery capabilities. The five cities with higher evaluation indices for this capacity are Zhengzhou, Jiyuan, Jiaozuo, Luoyang, and Sanmenxia, which benefit from higher government fiscal revenues, *per capita* disposable income, and *per capita* GDP, as well as a more significant proportion of primary medical insurance participation. These factors are key drivers of their more substantial post-emergency recovery capabilities.

#### 4.3.6 Learning capability

**Figure 9:**
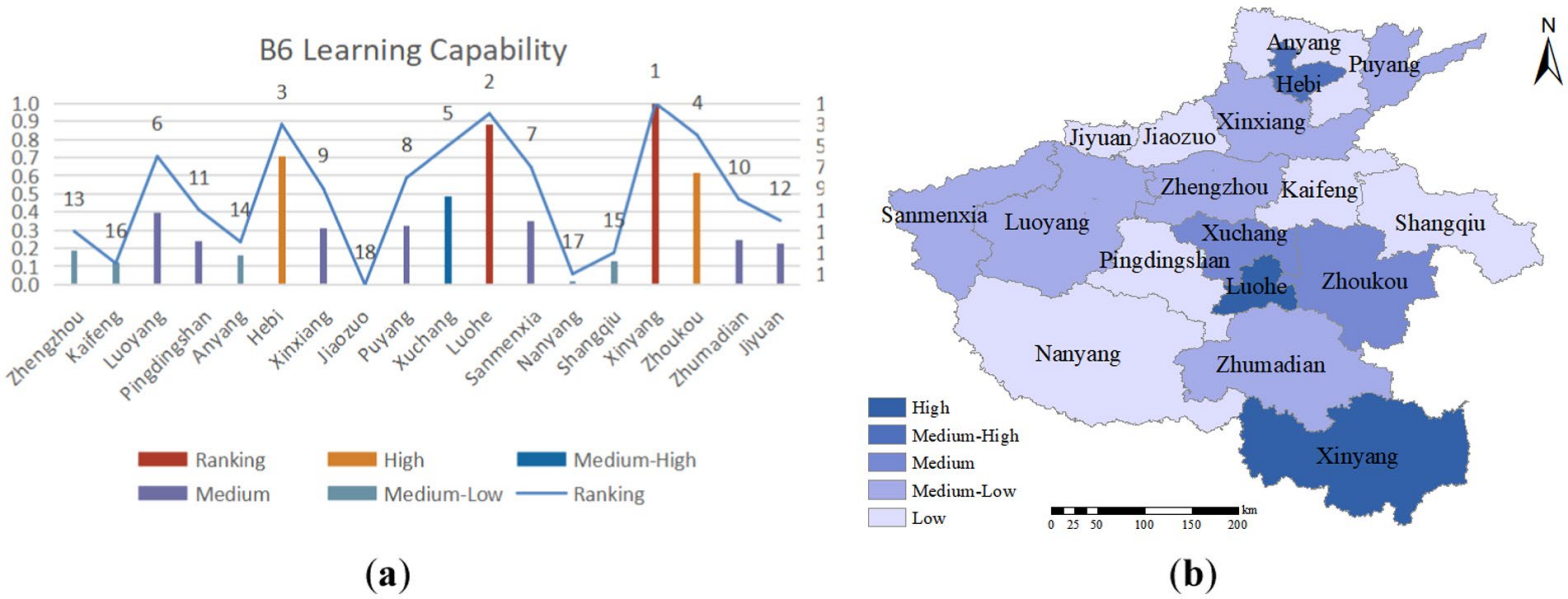
**(A)** Learning capability evaluation value and ranking; **(B)** learning capability assessment distribution.

The term “learning capability” refers to a city’s capacity to promptly and effectively conduct post-disaster summarization and learning, and to enhance its emergency response level through training and drills following public health emergencies. This metric is contingent upon two factors: the frequency of annual disaster prevention and reduction training, publicity, and drills and the frequency of public health emergencies that have occurred within the past five years. As illustrated in Figure 9, Xinyang, Luohe, and Hebi exhibit strong post-disaster learning abilities, indicating that they have experienced relatively fewer public health emergencies and have conducted more disaster prevention and reduction training. However, Anyang and Luoyang, owing to their geographical location and high population mobility, have encountered relatively more public health emergencies, which have provided valuable experience for local emergency management work but have also presented significant pressure and challenges.

## 5 Conclusion and suggestion

Drawing on the integration of the whole-process equilibrium theory and emergency management theory, this study presents an evaluation model for the emergency management capability of urban public health emergencies. The model comprises 31 indicators across six dimensions and is validated using data from 18 cities in Henan Province. The evaluation results reveal that Jiaozuo, Hebi, Zhengzhou, and Luohe exhibit high overall emergency management capability, while Kaifeng, Pingdingshan, and Shangqiu demonstrate relatively low capability. Furthermore, the northern region of Henan displays more robust emergency preparedness and early warning capabilities, whereas the central region exhibits more vital emergency mitigation and recovery capabilities. The study also finds that the emergency management capability of urban public health emergencies is closely linked to the frequency of disaster prevention and mitigation training, the proportion of financial investment in disaster prevention and emergency management, and the investment in medical resources. These conclusions provide a qualitative basis for the long-term layout of government emergency management in the later period. Based on these findings, the study proposes several strategies to enhance the emergency management capability of urban public health emergencies:

(1) Consolidate the legal review mechanism of public health emergency, and lay the legal foundation for improving the government’s emergency management ability (34). The legislature should fully implement the concepts of “big hygiene” and “big health,” firmly establish the basic idea of “constant change and constant innovation,” establish an efficient legal review mechanism, and ensure that public health laws keep up with the development trend of public health. Therefore, the disease control agencies and health professionals should learn the frontier knowledge in the field of social public health in real time, and review the professional knowledge related to the law of public health emergencies in a timely manner, and establish inter-departmental coordination mechanism, in order to review the law in a coordinated manner, to avoid conflicts or inconsistencies between different systems.
(2) Improve the planning of emergency medical facilities and enhance the reserve force of the government’s emergency preparedness capacity. The space reservation planning of emergency medical facilities can ensure that sufficient space and equipment are prepared for later emergency disposal in the event of damage or large-scale medical assistance. For example, the ‘shelter hospital’ during the COVID-19 epidemic has the advantages of short construction time, large capacity, low construction cost and high treatment efficiency. Emergency medical facilities can provide alternative locations in crisis situations. Therefore, cities should strengthen risk assessment in land use planning according to the characteristics of local natural and social environment, and maintain the accessibility of resources such as transportation, electricity, communication and water to meet the emergency needs of severe public health emergencies.
(3) Establish a diverse collaborative network to attract participation from all sectors of society. The remarkable achievements in epidemic prevention and control in China since the outbreak of COVID-19 are attributed to the governance concept of Party and government leadership, which relies on administrative power to coordinate the interests of residents, enterprises, and social organizations and construct an emergency management mechanism with multi-party participation (35). Therefore, to fully mobilize the participation of social organizations, the Red Cross, volunteer organizations, and news media, a diverse collaborative network should be established based on the development characteristics of each city and the local emergency management situation. This network should leverage different entities’ technical and professional advantages, enhancing the scientific and professional level of urban emergency management.
(5) Establish a smooth, sensitive information management system and strengthen public opinion guidance and supervision. Following the relevant policies of the state on big data, the government should solve the problem of information silos, ensuring the maximization of data value while also being vigilant against the leakage of private health information and its unauthorized use for commercial purposes. The government should guide public opinion to prevent the spread of false information and avoid amplifying people’s panic. A scientific system for public opinion management assessment and accountability should be established, and relevant management personnel should be held accountable for uncontrollable public opinion incidents.
(6) Based on reality, attach importance to post-disaster emergency management capacity assessment and learning. Cities should regard emergency management assessment as an essential indicator for evaluating grassroots administrative units. A system and mechanism for assessing and evaluating the prevention of sudden public health incidents that serve residents should be established, ensuring that every aspect of the prevention and control task of sudden public health incidents is traceable. After sudden public health incidents, the crisis should be transformed into an opportunity for development. Shortcomings exposed in the process of emergency rescue and control should be summarized and improved promptly, and multiple solutions should be proposed for various problems to prevent the recurrence of similar problems.

## Data Availability

The original contributions presented in the study are included in the article/supplementary material, further inquiries can be directed to the corresponding author.
